# Diversity, Leaf Anatomy, and Conservation of Fabaceae in a Thai Limestone Area

**DOI:** 10.3390/plants15172605

**Published:** 2026-08-26

**Authors:** Wichuda Manorin, Ponprom Pisuttimarn, Suparman Suparman, Henrik Balslev, Witsanu Saisorn

**Affiliations:** 1 Department of Biology, Section for Ecoinformatics & Biodiversity, Aarhus University, Ny Munkegade 114-116, DK-8000 Aarhus, Denmark; 202502090@post.au.dk (W.M.); henrik.balslev@bio.au.dk (H.B.); 2 Queen Sirikit Botanic Garden, The Botanical Garden Organization, Chiang Mai 50180, Thailand; bookponpisut@gmail.com; 3 Department of Biology Education, Faculty of Teacher Training and Education, Universitas Khairun. Jl. Pertamina Kampus II Unkhair Gambesi, Ternate 97728, North Maluku, Indonesia; suparman@unkhair.ac.id; 4 School of Science, Walailak University, Thasala, Nakhon Si Thammarat 80161, Thailand

**Keywords:** biodiversity, leaf epidermis, legumes, Leguminosae, micromorphology

## Abstract

Fabaceae, the third-largest family of angiosperms, occurs in diverse habitats, including limestone areas characterized by karst landscapes, and in Thailand, by frequent human disturbance due to activities such as agriculture and mining. These disturbances affect plant species, which highlights the importance of diversity studies and the use of morphology and anatomy for identification. A study of Fabaceae diversity in a limestone area in Thung Song District, southern Thailand, were conducted along three survey routes. A total of 23 genera and 30 species were encountered, including 2 vulnerable, 1 rare, and 8 introduced species. Morphological, phenological, ecological, and conservation status data were recorded. The leaf epidermis of 23 species was observed under a light microscope. Stomatal size and stomatal index were measured. *Phanera tubicalyx*, an endemic and vulnerable species restricted to limestone habitats, had multiple papillae on its epidermal cells, which may reduce water loss and enhance photosynthetic efficiency. Both hypostomatic and amphistomatic leaves were observed. Stomatal size ranged from 114.63 ± 17.57 μm^2^ to 608.88 ± 16.42 μm^2^, while the stomatal index varied from 0.85 ± 0.78 to 23.86 ± 0.16. Peculiar paired crystals and multiple papillae were found in *Canavalia cathartica* and *P. tubicalyx*, respectively. These and other morphological and leaf epidermal characteristics were useful for species identification. Human disturbances from agriculture and mining were common in the study area. In conclusion, this study documented 30 Fabaceae species and demonstrated the usefulness of morphological and leaf epidermal characteristics for species identification and conservation.

## 1. Introduction

Fabaceae, commonly referred to as the legume or pea family, is the third-largest family of angiosperms, following Asteraceae and Orchidaceae [1]. It comprises approximately 765 genera and about 19,500 species distributed worldwide. It is characterized by a superior, unicarpellate ovary with marginal placentation, which forms the characteristic legume fruit. Based on phylogenetic analyses of the *mat*K gene and morphological characteristics, Fabaceae is now divided into six subfamilies: Caesalpinioideae, Cercidoideae, Detarioideae, Duparquetioideae, Dialioideae, and Papilionoideae [1,2]. The subfamilies are recognized by their glandular structures on petal margins, flower type, seed hilar valve, embryo radicle, leaf type, extrafloral nectaries, stipule type, and inflorescence structure [1]. The family is one of the most ecologically and economically important families of flowering plants, playing key roles in natural ecosystems through nitrogen fixation while providing many of the world’s major food crops, such as the faba bean (*Vicia faba* L.), dry pea (*Lathyrus oleraceus* Lam.), soybean (*Glycine max* (L.) Merr.), chickpea (*Cicer arietinum* L.), and the common bean (*Phaseolus vulgaris* L.) [1,3,4,5,6]. Despite its global importance, many regional floras remain incompletely documented. In Thailand, Fabaceae includes 130 genera and over 600 species [7,8,9]. The Papilionoideae in the country are now being studied and generic treatments have already appeared, such as those of *Campylotropis* [10], *Codariocalyx*, *Dendrolobium*, *Hylodesmum*, *Ototropis*, *Phyllodium*, *Pleurolobus*, and *Sohmaea* [11,12,13,14,15,16]. A study of Fabaceae in Thailand shows that peninsular Thailand has the highest diversity of papilionoid legume species, with 63 genera and 199 species. *Crotalaria* is the most diverse genus in this floristic region [17,18]. Although numerous taxonomic revisions have clarified the diversity of several papilionoid legume genera, these studies have primarily focused on regional floras rather than specific habitats. Consequently, the diversity of Fabaceae in limestone ecosystems remains poorly documented. In addition to external morphological characteristics, leaf epidermal characteristics have been increasingly used as characteristics for species identification and delimitation. Features such as epidermal cell shape and arrangement, stomatal type and distribution, trichomes, papillae, and crystals can provide diagnostic information and are particularly useful for distinguishing morphologically similar taxa [11,12,13]. However, the application of leaf epidermal characteristics in the identification of Fabaceae species from Thai limestone habitats remains poorly documented.

Limestone habitats are among the most distinctive terrestrial ecosystems. Their shallow soils, exposed rock surfaces, and heterogeneous microhabitats promote plant specialization and endemism [19]. In Thailand, these habitats cover approximately two million hectares and are found throughout the country, with the largest areas located in Kanchanaburi, Tak, Lamphun, Chiang Mai, Mae Hong Son, and Saraburi provinces [20]. The country harbors around 800 endemic plant species, and of these, 180 are restricted to limestone habitats [21]. For example, the Phu Kieo–Nam Nao limestone area in northeastern Thailand has high plant diversity, with 22 families and 80 species of vascular plants, including 2 legume species. However, the total number of vascular plants in this area is estimated to comprise approximately 500 species and the Fabaceae is expected to be the second-largest family [20]. Despite the documented diversity of limestone flora, comprehensive inventories of Fabaceae diversity and studies of diagnostic leaf epidermal characteristics in these habitats remain scarce. Consequently, both the species diversity of Fabaceae and the potential taxonomic value of morphological and leaf epidermal characteristics in Thai limestone habitats remain insufficiently understood. Recent discoveries of new legume species from Thai limestone regions, including *Derris solorioides* Sirich. & Adema from Nakhon Sawan province [22], *Erythrina calcicola* Tetsana & Poopath from Loei province [23], and *Sophora huamotensis* Mattapha, Suddee & Rueangr. from Tak province [24], further highlight the taxonomic and floristic importance of these habitats.

Increasing human activities, particularly limestone mining, threaten limestone plant communities and can alter vegetation composition, species abundance, and species richness [25,26,27]. In Thailand, limestone mining has been a long-standing economic activity and a potential driver of plant biodiversity loss. Because many limestone species have restricted distributions, habitat destruction may lead to species loss before they are scientifically documented. Despite the extensive limestone landscapes of Thung Song District in southern Thailand, its Fabaceae flora has not been comprehensively investigated. This study hypothesizes that morphological and leaf epidermal characteristics provide additional diagnostic features for distinguishing Fabaceae species in limestone habitats. It is expected that this study will document a diverse Fabaceae flora, including species of conservation significance. Accordingly, this study aims to (1) produce an inventory of Fabaceae growing in the limestone area of Thung Song District; (2) investigate morphological and leaf epidermal characteristics for plant identification, thereby contributing taxonomic and systematic knowledge of the Thai flora; and (3) provide baseline data to support the conservation and management of limestone Fabaceae and other threatened plant species.

## 2. Results

### 2.1. Diversity, Morphology, and Key to Species

**Figure 1:**
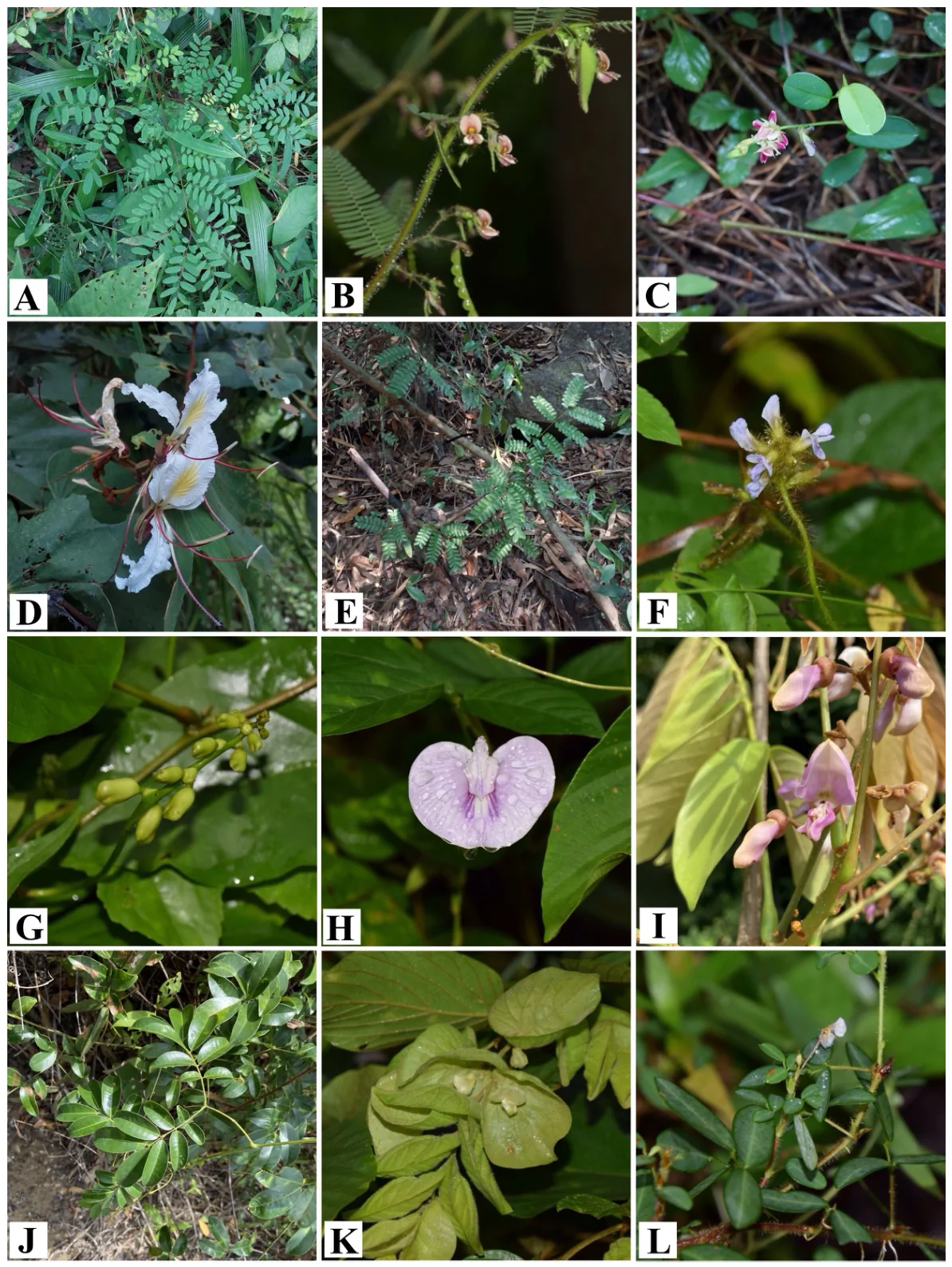
Selected species of Fabaceae in the Thung Song limestone area in southern Thailand. (**A**). *Adenanthera pavonina* (Manorin et al. 31), (**B**). *Aeschynomene americana* (Manorin et al. 11), (**C**). *Alysicarpus vaginalis* (Manorin et al. 21), (**D**). *Bauhinia pottsii* var. *subsessilis* (Manorin et al. 9), (**E**). *Biancaea parviflora* (Manorin et al. 39), (**F**). *Calopogonium mucunoides* (Manorin et al. 22), (**G**). *Canavalia cathartica* (Manorin et al. 13), (**H**). *Centrosema molle* (Manorin et al. 1), (**I**). *Derris elliptica* (Manorin et al. 33), (**J**). *Entada rheedei* (Manorin et al. 36), (**K**). *Flemingia strobilifera* (Manorin et al. 12), and (**L**). *Grona heterophylla* (Manorin et al. 16). ((**B**,**F**,**G**,**H**,**K**,**L**) taken by J. Satthaphorn; I taken by W. Saisorn; (**A**,**C**,**D**,**E**,**J**) taken by W. Manorin).

**Figure 2:**
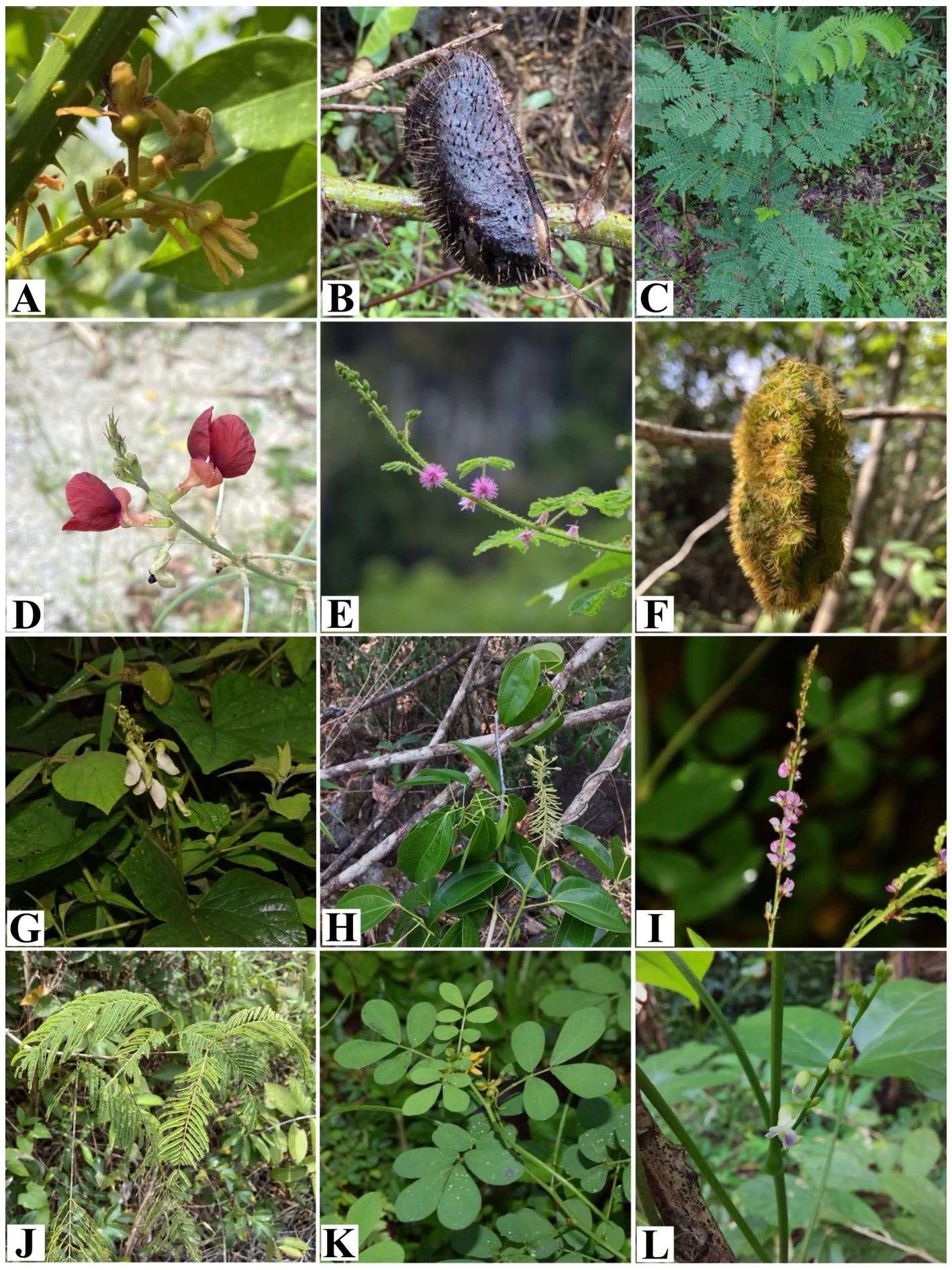
Selected species of Fabaceae in the Thung Song limestone area in southern Thailand. (**A**,**B**). *Guilandina major* (Manorin et al. 34), (**C**). *Leucaena leucocephala* (Manorin et al. 41), (**D**). *Macroptilium lathyroides* (Manorin et al. 32), (**E**). *Mimosa diplotricha* (Manorin et al. 20), (**F**). *Mucuna stenoplax* (Manorin et al. 37), (**G**). *Neustanthus phaseoloides* (Manorin et al. 19), (**H**). *Phanera tubicalyx* (Manorin et al. 38), (**I**). *Pleurolobus gangeticus* (Manorin et al. 18), (**J**). *Senegalia megaladena* (Manorin et al. 35), (**K**). *Senna tora* (Manorin et al. 15), and (**L**). *Teramnus labialis* (Manorin et al. 5). ((**E**,**G**,**I**,**K**) taken by J. Satthaphorn; (**A**,**B**,**D**,**F**,**J**) taken by W. Saisorn; (**C**,**H**,**L**) taken by W. Manorin).

A total of 23 genera and 30 species of Fabaceae were recorded in Thung Song limestone area in southern Thailand (Figure 1 and Figure 2, Table 1). Among these, *Phanera tubicalyx* (Craib) Bandyop. & Ghoshal was endemic to Thailand and classified as a vulnerable species, while *Flemingia strobilifera* (L.) W.T.Aiton was also considered vulnerable. In contrast, *Mucuna stenoplax* Wilmot-Dear was a rare species. Eight of the encountered Fabaceae species were introduced into Thailand, including *Aeschynomene americana* L., *Calopogonium mucunoides* Desv., *Centrosema molle* Mart. ex Benth., *Leucaena leucocephala* (Lam.) de Wit, *Macroptilium lathyroides* (Benth.) Urb., *Mimosa diplotricha* C.Wright, *M. pudica* L., and *Senna tora* (L.) Roxb. In addition, one species of *Phanera* could not be identified due to the absence of flowers and fruits. The morphological characteristics, phenology, conservation status, habitats, and specimens examined are summarized in Table 1 and Supplementary Table S1, and a key to species is provided below.
**Key to species of Fabaceae in Thung Song limestone area in southern Thailand**(based on morphology)1. Plants spinose or prickled. .............................................................................................................. 21. Plants not spinose or prickled. ....................................................................................................... 82. Leaf gland present. .......................................................................................................................... 42. Leaf gland absent. ............................................................................................................................ 33. Leaflets sessile. ..................................................................................................... *Biancaea parviflora*3. Leaflets petiolulate. ................................................................................................ *Guilandina major*4. Herbaceous. ...................................................................................................................................... 54. Scandent or woody climber. ........................................................................................................... 65. Leaves subdigitately compound, pinnae 1–2 pairs. ................................................ *Mimosa pudica*5. Leaves pinnately compound, pinnae more than 2 pairs. ................................ *Mimosa diplotricha*6. Pod distinctly wrinkled, indehiscent, transversely detached. ............................ *Senegalia rugata*6. Pod not wrinkled, dehiscent. .......................................................................................................... 77. The lowest petiolar gland at or above the middle of petiolar length. ....... *Senegalia megaladena*7. The lowest petiolar gland below the middle of petiolar length. ........................... *Senegalia torta*8. Leaf simple. ....................................................................................................................................... 98. Leaf unifoliolate, trifoliolate, or digitately, pinnately, or bipinnately compound. ................. 109. Climber; stem tendrils present. .................................................... *Phanera tubicalyx* & *Phanera* sp.9. Shrub to small tree; stem tendrils absent. ..................................... *Bauhinia pottsii* var. *subsessilis*10. Flower actinomorphic. ................................................................................................................. 1110. Flower zygomorphic or irregular. .............................................................................................. 1311. Climber; leaf tendril present. ................................................................................... *Entada rheedei*11. Tree; leaf tendril absent. .............................................................................................................. 1212. Inflorescences in spikes or compound spikes. .......................................... *Adenanthera pavonina*12. Inflorescences in spheric capitula or heads. .............................................. *Leucaena leucocephala*13. Stamens free. ................................................................................................................................. 1413. Stamens connate. .......................................................................................................................... 1514. Tree; leaf gland absent; fertile stamens 10. ....................................................... *Senna timoriensis*14. Herb; leaf gland present; fertile stamens 7. .................................................................. *Senna tora*15. Leaflets 1 or 3. ............................................................................................................................... 1715. Leaflets more than 5. .................................................................................................................... 1616. Erect herb. ................................................................................................. *Aeschynomene americana*16. Woody climber. ......................................................................................................... *Derris elliptica*17. Leaflet 1. ........................................................................................................................................ 1817. Leaflets 3. ....................................................................................................................................... 2018. Herbs; pod terete or laterally flattened. ..................................................................................... 1918. Shrub; pod turgid. .......................................................................................... *Flemingia strobilifera*19. Pod terete. ....................................................................................................... *Alysicarpus vaginalis*19. Pod laterally flattened. ................................................................................ *Pleurolobus gangeticus*20. Pod terete or laterally flattened. ................................................................................................. 2120. Pod turgid. .................................................................................................... *Flemingia macrophylla*21. Irritant bristles of fruits present. ....................................................................... *Mucuna stenoplax*21. Irritant bristles of fruits absent. .................................................................................................. 2222. Twining or climbing herbs. ......................................................................................................... 2522. Erect or prostrate herbs. .............................................................................................................. 2323. Flower zygomorphic; pod articulate. ......................................................................................... 2423. Flower asymmetric; pod not articulate. ................................................. *Macroptilium lathyroides*24. Pedicel 7–8 mm long. ................................................................................................. *Grona triflora*24. Pedicel longer than 8 mm. ................................................................................ *Grona heterophylla*25. Pod less than 1.5 cm wide. ........................................................................................................... 2625. Pod more than 1.5 cm wide. ........................................................................... *Canavalia cathartica*26. Stem covered with densely, erect ferruginous hairs. ...................... *Calopogonium mucunoides*26. Stem covered with pilose to densely appressed yellow hairs. ............................................... 2727. Standard petal longer than 1.5 cm. ............................................................................................ 2827. Standard petal shorter than 0.5 cm. ................................................................... *Teramnus labialis*28. Inflorescences usually small, less than 10 cm long. ......................................... *Centrosema molle*28. Inflorescences usually large, 10–50 cm long. ....................................... *Neustanthus phaseoloides*

### 2.2. Leaf Epidermis

#### 2.2.1. Epidermal Cells

**Figure 3:**
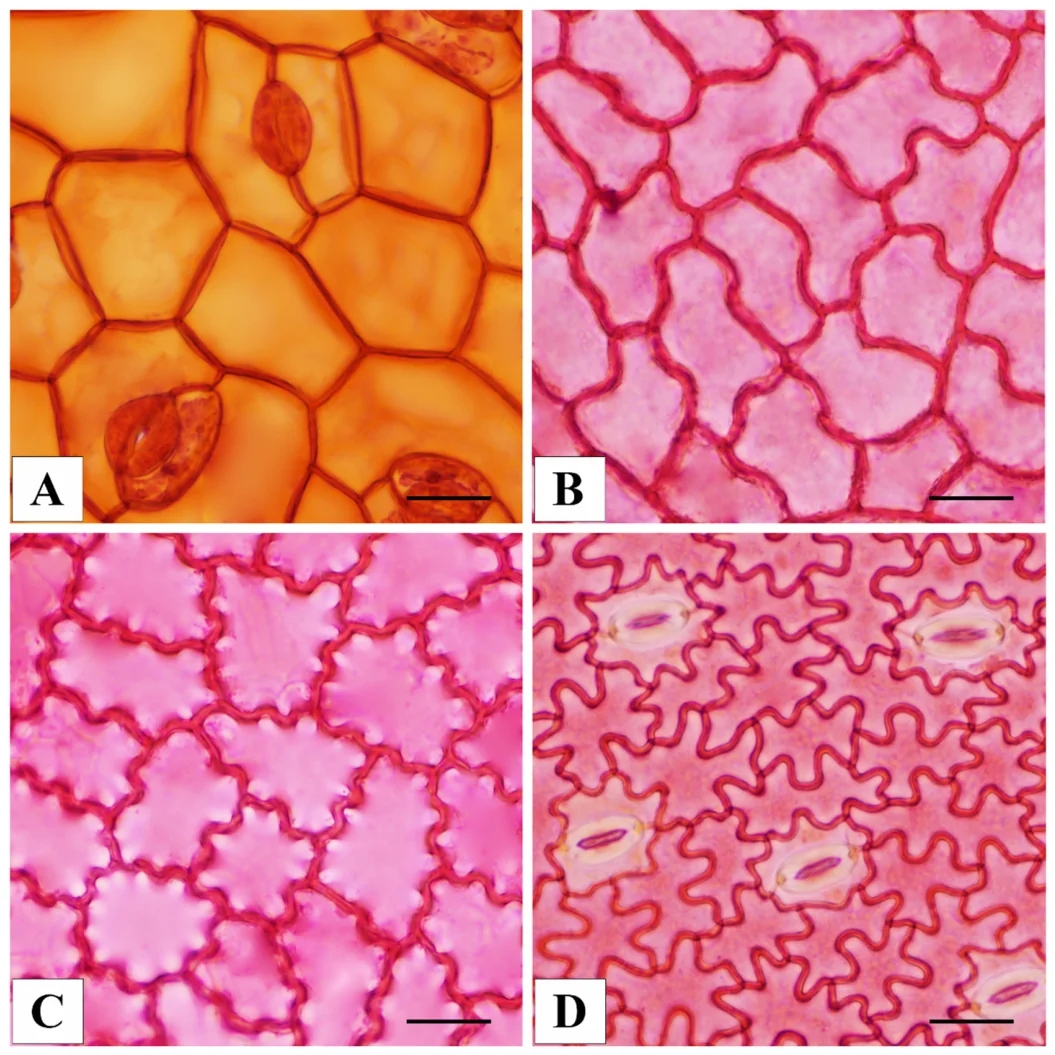
Epidermal cell types of Fabaceae in the Thung Song limestone area in southern Thailand. (**A**). Polygonal cells with straight-arched walls (adaxial epidermis of *Senna tora*, Manorin et al. 15); (**B**). Irregular cells with undulating walls (adaxial epidermis of *Derris elliptica*, Manorin et al. 33); (**C**). Jigsaw-like cells with sinuolate walls (adaxial epidermis of *Biancaea parviflora*, Manorin et al. 39); (**D**). Jigsaw-like cells with sinuate walls (abaxial epidermis of *Senegalia torta*, Manorin et al. 6). Scale bars 20 μm.

*Adaxial epidermis:* The adaxial epidermis was most commonly composed of jigsaw-like epidermal cells, which was observed in 18 species (Figure 3C,D), followed by irregular cells in eight species (Figure 3B). A smaller group—*Alysicarpus vaginalis*, *Entada rheedei*, *Guilandina major*, *Phanera tubicalyx*, and *Senna tora*—had polygonal cells (Figure 3A). In eight species, the epidermis showed variation in cell shape, and was composed of jigsaw-like cells combined with either irregular or polygonal cells. The anticlinal cell walls of epidermal cells exhibited a range of wavy patterns, ranging from very fine to strongly undulating. These included sinuolate, sinuate, and undulate types, which were predominantly associated with jigsaw-like and irregular cells. In contrast, straight-arched anticlinal walls represented a distinct pattern and were mainly found in species with polygonal cells (Figure 3A). Three species, *A. vaginalis*, *Grona triflora*, and *P. tubicalyx*, had papillae on the outer periclinal walls of their epidermal cells; the papillae were either single or multiple on each cell. A peculiar type of paired crystals in the epidermal cells was observed in *Canavalia cathartica*. *Abaxial epidermis:* The abaxial epidermis of most species was composed of irregular cells, but a limited number of species—*A. vaginalis*, *E. rheedei*, *P. tubicalyx*, and *S. tora*—had polygonal abaxial epidermal cells, largely similar to the adaxial epidermis. Anticlinal wall patterns of abaxial epidermal cells were diverse and included sinuate, sinuolate, straight-arched, and undulate types. A type with single papillae on the outer periclinal wall of epidermal cells was observed in *A. vaginalis* and *G. triflora*. Peculiar paired crystals were found in the epidermal cells of *C. cathartica* (Table 2).

#### 2.2.2. Stomata

**Figure 4:**
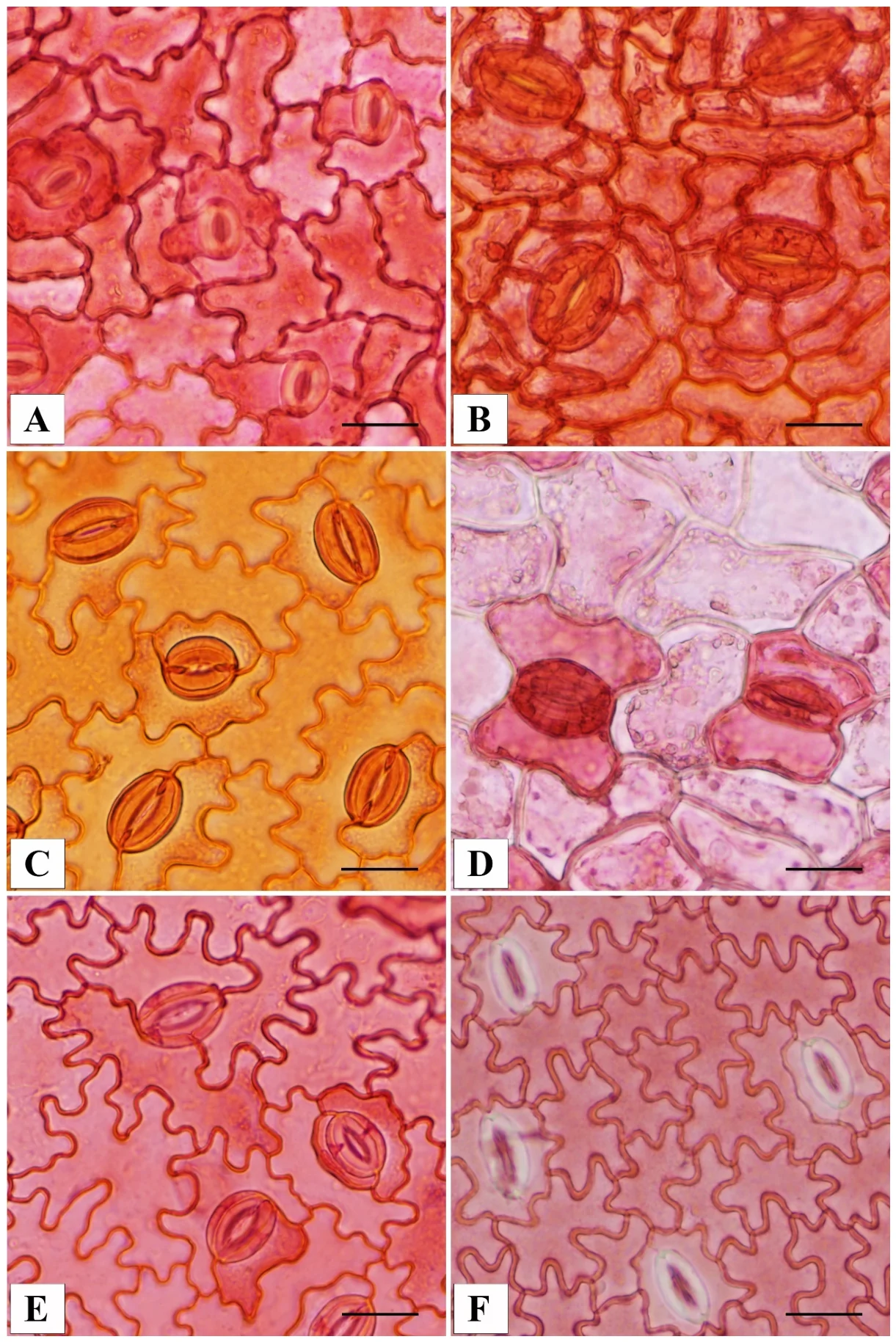
Stomatal types of Fabaceae in the Thung Song limestone area in southern Thailand. (**A**). Anisocytic stomata (abaxial epidermis of *Biancaea parviflora*, Manorin et al. 39); (**B**). Anomocytic stomata (abaxial epidermis of *Guilandina major*, Manorin et al. 34); (**C**). Laterocytic stomata (abaxial epidermis of *Calopogonium mucunoides*, Manorin et al. 22); (**D**). Paracytic stomata (abaxial epidermis of *Entada rheedei*, Manorin et al. 36); (**E**). Anomocytic and laterocytic stomata (abaxial epidermis of *Flemingia strobilifera*, Manorin et al. 3); (**F**). Laterocytic and paracytic stomata (abaxial epidermis of *Senegalia torta*, Manorin et al. 6). Scale bars 20 μm.

*Adaxial stomata:* Leaves of ten species—*Adenanthera pavonina*, *Biancaea parviflora*, *Derris elliptica*, *E. rheedei*, *G. major*, *Mucuna stenoplax*, *P. tubicalyx*, *Pleurolobus gangeticus*, *Senegalia torta*, and *Teramnus labialis*—were hypostomatic, whereas those of the remaining species were amphistomatic. The stomatal index ranged from 0.85 ± 0.78 in *Bauhinia pottsii* var. *subsessilis* to 18.02 ± 0.74 in *Aeschynomene americana*. The stomatal types included anomocytic, laterocytic, and paracytic, with sizes ranging from 114.63 ± 17.57 μm^2^ in *G. triflora* to 553.30 ± 122.36 μm^2^ in *C. cathartica*. The paracytic type observed in *E. rheedei* had a distinct feature, which was the symmetric, wide subsidiary cells that did not cover the ends of the guard cells. In contrast, *P. tubicalyx* had paracytic stomata with symmetric, narrow subsidiary cells that covered the ends of the guard cells. *Abaxial stomata:* Four stomatal types were observed on the abaxial epidermis: anisocytic (Figure 4A), anomocytic (Figure 4B), laterocytic (Figure 4C), and paracytic (Figure 4D). The abaxial paracytic stomata of both *E. rheedei* and *P. tubicalyx* were similar to those observed on their adaxial epidermis. Most species had mixed stomatal types; for example, *Flemingia strobilifera* (Figure 4E) and *S. torta* (Figure 4F). The stomatal index ranged between 7.26 ± 0.60 in *G. major* to 23.86 ± 0.16 in *A. americana*, while stomatal sizes ranged from 143.88 ± 0.68 μm^2^ in *G. triflora* to 608.88 ± 16.42 μm^2^ in *G. major* (Table 2).

#### 2.2.3. Trichomes

**Figure 5:**
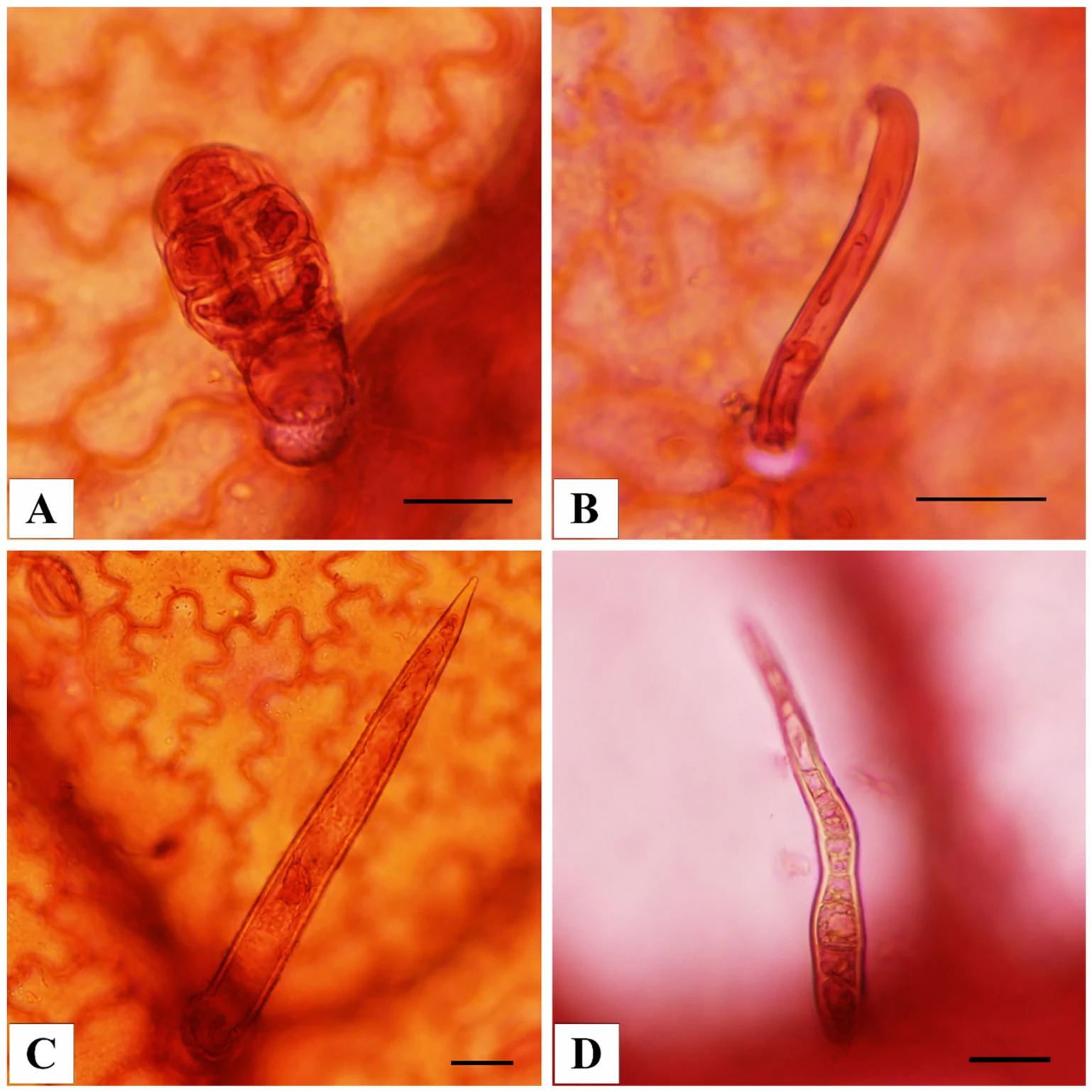
Trichome types of Fabaceae in Thung Song limestone area in southern Thailand. (**A**). Capitate glandular trichome (adaxial epidermis of *Teramnus labialis*, Manorin et al. 5); (**B**). Hooked trichome (adaxial epidermis of *Pleurolobus gangeticus*, Manorin et al. 18); (**C**). Unicellular trichome (adaxial epidermis of *Calopogonium mucunoides*, Manorin et al. 22); (**D**). Multicellular uniseriate trichome (adaxial epidermis of *Bauhinia pottsii* var. *subsessilis*, Manorin et al. 9). Scale bars 20 μm.

*Adaxial epidermis:* Trichomes were present on the adaxial epidermis of 11 species in our sample. The types of trichomes included capitate glandular (Figure 5A), hooked (Figure 5B), unicellular (Figure 5C), papillae, and multicellular uniseriate (Figure 5D). *Abaxial epidermis:* Trichomes were more frequent on the abaxial epidermis and included capitate glandular, hooked, unicellular, papillae, and multicellular uniseriate types. Trichomes were absent in five species: *A. pavonina*, *A. americana*, *B. parviflora*, *E. rheedei*, and *Leucaena leucocephala* (Table 2).

#### 2.2.4. Identification Key Using Anatomical Characters


**Key to species**
(based on leaf epidermis)1. Leaves hypostomatic. .................................................................................................................................................................................................................................................................. 21. Leaves amphistomatic. .............................................................................................................................................................................................................................................................. 112. Trichomes present on adaxial epidermis. ................................................................................................................................................................................................................................. 32. Trichomes absent on adaxial epidermis. ................................................................................................................................................................................................................................... 63. Capitate trichomes present on adaxial epidermis. ................................................................................................................................................................................................................... 43. Capitate trichomes absent on adaxial epidermis. .................................................................................................................................................................................................................... 54. Unicellular trichomes present on adaxial epidermis. .................................................................................................................................................................................... *Teramnus labialis*4. Unicellular trichomes absent on adaxial epidermis. .................................................................................................................................................................................... *Mucuna stenoplax*5. Hooked trichomes present on adaxial epidermis; capitate trichomes present on abaxial epidermis. ............................................................................................ *Pleurolobus gangeticus*5. Hooked trichomes absent on adaxial epidermis; capitate trichomes absent on abaxial epidermis. ............................................................................................................ *Senegalia torta*6. Trichomes present on abaxial epidermis. ................................................................................................................................................................................................................................. 76. Trichomes absent on abaxial epidermis. ................................................................................................................................................................................................................................... 87. Epidermal cell on adaxial epidermis mostly polygonal, rarely mixed with irregular; Stomata on abaxial epidermis anomocytic; stomatal index of abaxial epidermisless than 10. ............................................................................................................................................................................................................................................................ *Guilandina major*7. Epidermal cell on adaxial epidermis jigsaw-like and irregular; stomata on abaxial epidermis laterocytic and paracytic; stomatal index of abaxial epidermis more than 10. .............................................................................................................................................................................................................................................................. *Derris elliptica*8. Epidermal cell on adaxial epidermis jigsaw-like only. ........................................................................................................................................................................................................... 98. Epidermal cell on adaxial epidermis mostly polygonal, rarely mixed with jigsaw-like. .................................................................................................................................................. 109. Stomata on abaxial epidermis anisocytic. .................................................................................................................................................................................................... *Biancaea parviflora*9. Stomata on abaxial epidermis paracytic. ................................................................................................................................................................................................ *Adenanthera pavonina*10. Outer periclinal walls of epidermal cell on adaxial epidermis with multiple papillae. ......................................................................................................................... *Phanera tubicalyx*10. Outer periclinal walls of epidermal cell on adaxial epidermis without papillae. ........................................................................................................................................ *Entada rheedei*11. Trichomes present on adaxial epidermis. ............................................................................................................................................................................................................................. 1211. Trichomes absent on adaxial epidermis. ............................................................................................................................................................................................................................... 1912. Epidermal cell on adaxial epidermis polygonal; outer periclinal walls of epidermal cell on both adaxial and abaxial epidermis with single papillae. ......... *Alysicarpus vaginalis*12. Epidermal cell on adaxial epidermis jigsaw-like or/and irregular; outer periclinal walls of epidermal cell on both adaxial and abaxial epidermis without single papillae. ... 1313. Epidermal cells of both adaxial and abaxial epidermal with peculiar, paired crystals. ..................................................................................................................... *Canavalia cathartica*13. Epidermal cells of both adaxial and abaxial epidermis without peculiar, paired crystals. ............................................................................................................................................ 1414. Anticlinal wall of epidermal cells on adaxial epidermis undulate; Trichomes on both adaxial and abaxial epidermis multicellular uniseriate. .... *Bauhinia pottsii* var. *subsessilis*14. Anticlinal wall of epidermal cells on adaxial epidermis sinuate; trichomes on both adaxial and/or abaxial epidermis capitate and/or unicellular. ............................................ 1515. Capitate trichomes present on abaxial epidermis. .............................................................................................................................................................................................................. 1615. Capitate trichomes absent on both adaxial and abaxial epidermis. ....................................................................................................................................................... *Mimosa diplotricha*16. Both capitate and unicellular trichomes present on adaxial epidermis. ........................................................................................................................................................................... 1716. Only capitate trichomes present on adaxial epidermis. ........................................................................................................................................................................ *Flemingia strobilifera*17. Stomatal index of adaxial epidermis more than 5. .............................................................................................................................................................................................................. 1817. Stomatal index of adaxial epidermis less than 5. ......................................................................................................................................................................................... *Centrosema molle*18. Anomocytic and laterocytic stomata present on adaxial epidermis; stomatal index of adaxial epidermis more than 10. ................................................... *Calopogonium mucunoides*18. Anomocytic, laterocytic, and paracytic stomata present on adaxial epidermis; stomatal index of adaxial epidermis less than 10. .................................... *Neustanthus phaseoloides*19. Epidermal cell on adaxial epidermis jigsaw-like and/or irregular. ................................................................................................................................................................................... 2019. Epidermal cell on adaxial epidermis polygonal. ..................................................................................................................................................................................................... *Senna tora*20. Outer periclinal walls of epidermal cell on both adaxial and abaxial epidermis with single papillae. ....................................................................................................... *Grona triflora*20. Outer periclinal walls of epidermal cell on both adaxial and abaxial epidermis without papillae. ............................................................................................................................... 2121. Stomatal index of abaxial epidermis more than 20. ............................................................................................................................................................................................................. 2221. Stomatal index of abaxial epidermis less than 20. ................................................................................................................................................................................ *Leucaena leucocephala*22. Trichomes present on abaxial epidermis. ......................................................................................................................................................................................... *Macroptilium lathyroides*22. Trichomes absent on abaxial epidermis. .......................................................................................................................................................................................... *Aeschynomene americana*

## 3. Discussion

### 3.1. Diversity and Identification of Fabaceae in Thung Song Limestone Area

The three subfamilies of Fabaceae recorded in the area were Caesalpinioideae, Cercidoideae, and Papilionoideae, which included 12, 3, and 15 species, respectively. *Senegalia* is the genus with the highest numbers of species, and includes *S. megaladena*, *S. rugata*, and *S. torta*. These species are commonly found along roadsides and in evergreen forests of limestone mountains. In Thailand, *Senegalia* occurs in various habitats, particularly in mixed deciduous forest, including limestone areas. Species of this genus are fast growing and well adapted to a wide range of environmental conditions. Some species grow in nutrient-deficient sites and tolerate dry conditions [29,30]. These ecological traits may explain the occurrence of *Senegalia* species in the limestone habitats of the present study. Five species recorded in this study—*Aeschynomene americana*, *Calopogonium mucunoides*, *Centrosema molle*, *Mimosa pudica*, and *Neustanthus phaseoloides*—have also been reported from Khao Wong Phra Chan limestone area, Songkhla province, southern Thailand [17]. All species of Papilionoideae identified in the present study have previously been reported from southern Thailand, with four species—*C. molle*, *Flemingia macrophylla*, *Grona triflora*, and *Pleurolobus gangeticus*—known to occur in limestone habitats [18]. A total of 33 Fabaceae species have been reported from Namtok Ngao Waterfall national park, Ranong province, southern Thailand, of which five—*Adenanthera pavonina*, *Bauhinia pottsii* var. *subsessilis*, *Derris elliptica*, *Entada rheedei*, and *S. torta*—are shared with the present study [31]. These overlapping records suggest that Fabaceae diversity in the study area includes both widely distributed species in southern Thailand and more narrowly distributed species that are associated with limestone habitats. Additionally, two vulnerable species (*F. strobilifera* and *Phanera tubicalyx*) and one rare species (*Mucuna stenoplax*) were observed; the vulnerable and rare conservation statuses of these species were previously assessed by Chamchumroon et al. [28]. *Phanera tubicalyx*, found on a limestone peak in this study, is a species endemic to Thailand and restricted to limestone habitats [7]. This finding suggests that the limestone area in Thung Song District may provide important habitats for narrowly distributed Fabaceae species. *Mucuna stenoplax* has a narrow distribution, occurring in five provinces of southern Thailand and in Perlis, a bordering state of Malaysia [9]. Eight species recorded in this study were introduced to Thailand, including *A. americana*, *C. mucunoides*, *C. molle*, *Leucaena leucocephala*, *Macroptilium lathyroides*, *Mimosa diplotricha*, *M. pudica*, and *Senna tora* [7,8,18]. The occurrence of these introduced species, especially in disturbed habitats such as roadsides, is surely the result of human activities in parts of the study area.

Morphological characteristics were useful for plant identification, as shown on the key to species based on morphology. The spines and prickles found on stems and leaves were used to identify seven species, namely *Biancaea parviflora*, *Guilandina major*, *M. diplotricha*, *M. pudica*, *S. megaladena*, *S. rugata*, and *S. torta*. Habit, leaf type, leaf gland, pinnae, and pod surfaces were other characteristics important for identification of these species. The remaining 23 species had no spines or prickles. Three taxa were characterized by simple leaves and their growth habits, while other species had compound leaves. Species with compound leaves can be further distinguished by various characteristics of the stem, tendril, leaf, inflorescence, pedicel, petal, stamen, and pod.

### 3.2. Epidermal Cells

The leaf epidermis had considerable variation in epidermal cell shape and anticlinal wall patterns. Polygonal cells with straight-arched walls were relatively uncommon, being observed only in *Alysicarpus vaginalis*, *E. rheedei*, *G. major*, *P. tubicalyx*, and *S. tora*. This finding is consistent with previous reports by Souto et al. [32] and Begum et al. [33], who described straight to curved anticlinal walls of *Senna* species. Polygonal cells have also been widely documented in several legumes; for example, nine species of *Crotalaria* [34], fifteen species of *Oxytropis* [35], and one species of *Phyllodium* [11]. In addition, Pandey et al. [36] reported this cell shape as the most common epidermal characteristic in Caesalpinioideae, occurring in 16 out of 25 species examined. The irregular epidermal cells were recorded in 12 species in the present study, namely *A. americana*, *B. pottsii* var. *subsessilis*, *B. parviflora*, *Canavalia cathartica*, *D. elliptica*, *E. rheedei*, *G. triflora*, *G. major*, *L. leucocephala*, *M. lathyroides*, *P. gangeticus*, and *S. tora*. In contrast, Pandey et al. [36] reported, in their investigation of leaf epidermal characteristics across 14 genera and 25 species of Caesalpinioideae, that irregular epidermal cells were present in only a few species, including *Bauhinia acuminata* L., *Peltophorum pterocarpum* (DC.) Backer ex K.Heyne, *Phanera vahlii* (Wight & Arn.) Benth., *Senna obtusifolia* (L.) H.S.Irwin & Barneby, *S. occidentalis* (L.) Link, and *S. pallida* (Vahl) H.S.Irwin & Barneby. This cell shape has also been reported in other legume genera, including several species of *Crotalaria* and *Oxytropis*, as well as a limited number of species in *Codariocalyx* and *Phyllodium* [11,13,34,35]. In the present study, the most common epidermal cell shape, observed in nearly all species, was jigsaw like, except in *Alysicarpus vaginalis*, *E. rheedei*, and *S. tora*. This characteristic has been reported in several genera within the family, e.g., *Bauhinia*, *Caesalpinia*, *Codariocalyx*, *Crotalaria*, *Guilandina*, *Libidibia*, and *Phyllodium* [11,13,34,35,36]. This widespread occurrence of jigsaw-like epidermal cells suggests that this characteristic may be common within the family. The epidermal cell shape complexity observed in the studied species, including jigsaw-like pavement cells, may be associated with adaptation to environmental conditions, particularly water availability. This interpretation is consistent with Liu et al. [37], who suggested that pavement cell shape complexity in European aspen (*Populus tremula* L.) reflects both evolutionary adaptation to environmental water availability and developmental responses to local drought stress during leaf growth. Three types of wavy anticlinal wall patterns—sinuate, sinuolate, and undulate—were identified in this study. These patterns have also been reported in genera *Bauhinia*, *Caesalpinia*, *Codariocalyx*, *Crotalaria*, *Guilandina*, *Libidibia*, *Phyllodium*, and *Schnella* [11,13,34,35,36,38]. The present study also revealed variation in epidermal cell shape and/or anticlinal wall patterns on the same leaf surface within individual species, as observed in 15 species. Such variation has previously been reported in Fabaceae. For example, anticlinal walls of adaxial epidermal cells in *Cassia roxburghii* DC., *Lysiphyllum hookeri* (F.Muell.) Pedley, and *P. pterocarpum* can be either straight or undulate [36]. Similar variation has been documented in ten *Crotalaria* and two *Phyllodium* species from Thailand [11,34]. In contrast, *C. molle* in the present study showed no variation in epidermal cell shape or anticlinal wall patterns on the same leaf surface, whereas Chukwuma et al. [39] reported that this species has irregular and rectangular cells with straight, undulate, and undulate-cuneate anticlinal walls. The presence of peculiar paired crystals in epidermal cells, observed in this study, represents a distinct characteristic of *C. cathartica*, which has been previously reported in the genus *Canavalia* by Lackey [40]. Therefore, the presence of peculiar paired crystals may provide an additional useful characteristic for recognizing this genus, although further study is needed.

Our study suggested that characteristics of epidermal cells alone or combined with other characteristics were useful for plant identification, e.g., *G. major* was distinguished from *D. elliptica* by polygonal epidermal cells on the adaxial epidermis, anomocytic stomata on the abaxial epidermis, and a stomatal index value on the abaxial epidermis; peculiar paired crystals of epidermal cells were restricted to species mentioned above.

### 3.3. Stomata

The leaves of the studied species were either hypostomatic or amphistomatic. Hypostomatic leaves were found in ten species belonging to *Adenanthera*, *Biancaea*, *Derris*, *Entada*, *Guilandina*, *Mucuna*, *Phanera*, *Pleurolobus*, *Senegalia*, and *Teramnus*. The occurrence of hypostomatic leaves in some of those genera has been documented in previous studies (for example, in *Bauhinia* and *Phanera* [36]), as well as in *Derris* [41]. In the present study, one species of *Guilandina* had hypostomatic leaves, whereas Pandey et al. [36] reported amphistomatic leaves in another species within the same genus (*G. bonduc* L.). Furthermore, *M. stenoplax* had hypostomatic leaves; in contrast, two other species reported previously—*M. pruriens* (L.) DC. and *M. poggei* Taub.—were described as amphistomatic [42]. These differences suggest that stomatal distribution can vary among species within the same genus and may be used as a diagnostic characteristic between species in the same genus. The remaining species examined in this study were amphistomatic, a feature which has been widely reported across legume genera. For instance, Pandey et al. [36] reported amphistomatic leaves in 11 genera and 17 species within Caesalpinioideae. Similarly, Owolabi & Adedeji [43] documented this characteristic in seven genera and eight species of Papilionoideae in Nigeria. This feature has also been observed in studies focusing on a few genera or species [11,12,34,35,38,42,44]. The predominance of amphistomatic leaves among the studied Fabaceae species may reflect adaptation to the exposed conditions of limestone habitats. This interpretation is supported by Tkach et al. [45], who documented predominantly amphistomatic leaves and considerable interspecific variation in stomatal distribution among *Saxifraga* species (family Saxifragaceae), suggesting that stomatal distribution may have physiological and evolutionary adaptation significance.

We found four stomatal types: anisocytic, anomocytic, laterocytic, and paracytic. The most common type was laterocytic, followed by paracytic, while the rarest type was anisocytic, which was observed on the abaxial epidermis of *B. parviflora*. In contrast, anisocytic stomata have been reported as one of the more common types in Papilionoideae [43] and Fabaceae [44]. The result of the present study was consistent with that of Pandey et al. [36], who reported these stomatal types in Caesalpinioideae, along with three additional types—actinocytic, diacytic, and tetracytic. Among these, paracytic stomata were common, whereas the additional types were rarely observed in this subfamily. Anomocytic and anisocytic stomata have also been reported in the genera *Bauhinia*, *Crotalaria*, and *Schnella* [34,38]. Moreover, anisocytic and paracytic stomata have been documented in *Oxytropis* and *Derris* [35,41], while anomocytic and paracytic stomata have been reported in *Codariocalyx* and *Phyllodium* [11,12]. In *Mucuna*, stomatal types include anomocytic, anisocytic, and paracytic [42]. These comparisons indicate that stomatal type is variable within Fabaceae and serve as a useful characteristic for plant identification and classification. In the adaxial epidermis, the stomatal index ranged from 0.85% in *B. pottsii* var. *subsessilis* to 18% in *A. americana*. In contrast, the stomatal index of the abaxial epidermis was consistently higher across all studied species, ranging from 7% in *G. major* to 24% in *A. americana*. The stomatal index values observed in Papilionoideae of this study are consistent with those reported by Owolabi & Adedeji [43]. Furthermore, our results are largely in agreement with those of Pandey et al. [36], with the exception of seven species in their study in which the stomatal index of the adaxial epidermis exceeded that of the abaxial epidermis. Comparisons between the present study and Pandey et al. [36] suggest that the genus *Guilandina* may have a stomatal index that is relatively lower than that of other members of the family. However, this finding contrasts with Zhao et al. [35], who reported that in all examined *Oxytropis* species, the mean stomatal index of the adaxial epidermis was higher than that of the abaxial epidermis.

Here, stomatal distribution was the primary characteristic used to differentiate the studied species into two groups: one consisting of ten hypostomatic species and another comprising thirteen amphistomatic species. The stomatal index value combined with stomatal types separated *C. mucunoides* and *N. phaseoloides*, and the stomatal index alone was also used to distinguish *C. molle* and *L. leucocephala* from other species shown in the key to species based on leaf epidermis.

### 3.4. Trichomes

Trichome types were observed in 18 species, and they include a glandular type (capitate) or non-glandular types (hooked, unicellular, papillae, and multicellular uniseriate). Trichomes were absent in five species: *A. pavonina*, *A. americana*, *B. parviflora*, *E. rheedei*, and *L. leucocephala*. Glandular trichomes have been reported in several species across different tribes of Fabaceae; for example, Desmodieae, Diocleae, and Phaseoleae [46]. Our results are consistent with this report, as glandular trichomes were observed in species belonging to these tribes: seven species from Phaseoleae (*C. mucunoides*, *C. molle*, *F. strobilifera*, *M. lathyroides*, *M. stenoplax*, *N. phaseoloides*, and *Teramnus labialis*); three species from Desmodieae (*A. vaginalis*, *G. triflora*, and *P. gangeticus*); one species from Diocleae (*Canavalia cathartica*). Glandular trichomes were found not only on the leaf blade epidermis but also on the petiole epidermis, as previously reported in the genus *Flemingia* [47]. Multicellular uniseriate trichomes were restricted to two species, *B. pottsii* var. *subsessilis* and *S. tora*. Similarly, Begum et al. [33] reported this trichome type on the abaxial epidermis of *S. tora*. In the present study, hooked trichomes were restricted to three species of Desmodieae (*A. vaginalis*, *G. triflora*, and *P. gangeticus*). Metcalfe and Chalk [46] noted that this trichome type is common in several genera of Desmodieae (e.g., *Alysicarpus*, *Desmodium*, and *Uraria*), and also occurs in other tribes, such as Phaseoleae (*Centrosema*). Unicellular trichomes have also been widely reported across the family, such as in the genera *Bauhinia*, *Cassia*, *Delonix*, *Guilandina*, *Parkinsonia*, *Peltophorum*, *Phanera*, *Senna*, and *Teramnus* [36]. In the present study, two types of papillae—single and multiple—were observed on the outer periclinal walls of epidermal cells. Single papillae were observed in *A. vaginalis* and *G. triflora*, whereas multiple papillae were found in *P. tubicalyx*. Papillae on subsidiary cells are known to function in pathogen defense and regulation of water loss [48]. In addition, papillae may contribute to improved physiological performance, such as reducing water loss as well as increasing relative water content, water use efficiency, intercellular CO_2_ content, carboxylation efficiency, and net assimilation rate [49]. *Phanera tubicalyx* has previously been reported as a limestone-associated species endemic to Thailand [7], and its occurrence in limestone habitats was also confirmed in the present study. Multiple papillae in this species may represent an adaptation to limestone environments, possibly contributing to improved water regulation and stress tolerance. Similarly, García-Martínez et al. [50] documented abundant and well-developed leaf papillae in *Otatea* (family Poaceae) and examined leaf micromorphological variation in relation to xeric and mesic environments, supporting the potential ecological significance of epidermal papillae. Apart from stomatal distribution, trichome distribution and types were quite useful for identifying plants in this study. The presence or absence of trichomes, and four different types of trichome, were the key for identifying species within those two groups mentioned in the previous section.

### 3.5. Habitat Loss and Conservation Implications

Disturbance and reduction of natural habitats in the study area were observed during fieldwork, primarily in the form of agricultural and mining activities. Based on direct measurements of Google Maps (https://www.google.com/maps) satellite imagery, approximately 5.0 km^2^ (33.8%) of the estimated 14.8 km^2^ study area was affected by habitat alteration associated with these activities. Although these estimates are approximate and not based on systematic remote-sensing or field-based assessments, they provide an initial indication of anthropogenic modification of the limestone habitat and highlight the need for further spatial assessment and conservation planning. Mining activities can alter vegetation composition, plant species abundance, and richness [26,27], potentially increasing threats to vulnerable and rare species, such as *F. strobilifera, P. tubicalyx*, and *M. stenoplax* [28]. Additionally, *P. tubicalyx* is of conservation importance because it is endemic to Thailand and is specifically associated with limestone habitats [7]. Habitat specificity makes this species particularly vulnerable to disturbance. Mining, agricultural expansion, and other land uses could reduce its habitats and potentially increase the risk of local extinction in the wild. Accordingly, conservation efforts should therefore prioritize the protection of *P. tubicalyx*, followed by *F. strobilifera* and *M. stenoplax*, given their vulnerable and rare statuses, respectively. Management actions should therefore focus on reducing further habitat degradation and monitoring populations of endemic, vulnerable, and rare species.

## 4. Materials and Methods

### 4.1. Study Area

**Figure 6:**
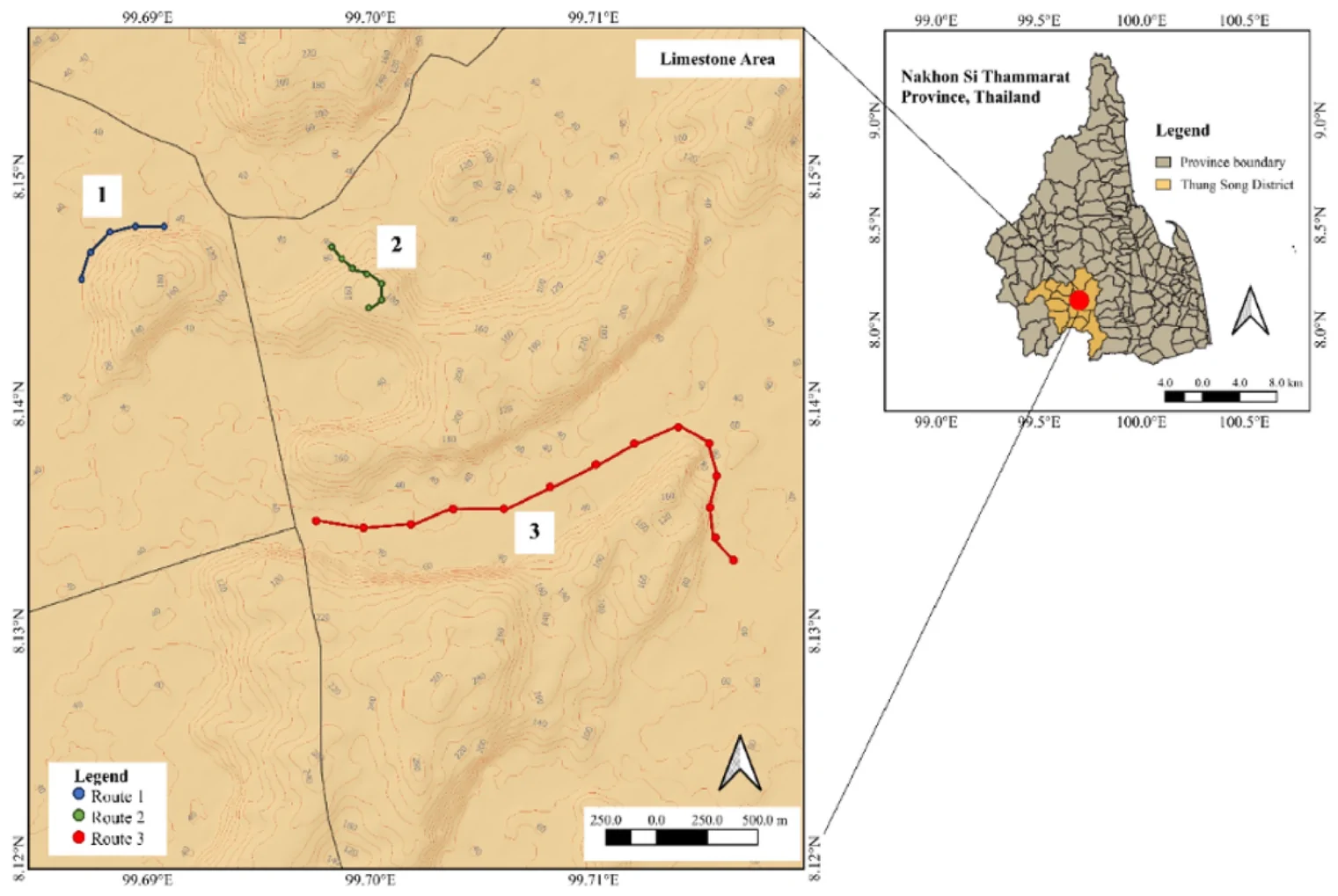
Study sites in Thung Song District, Nakhon Si Thammarat Province, in southern Thailand, showing the three survey routes. Map generated using QGIS software version 3.34.14.

Field surveys were conducted monthly in the limestone area of Thung Song District, Nakhon Si Thammarat Province, southern Thailand, from October 2022 to September 2023, with each survey lasting 1–2 days. The total study area encompassed 14.80 km^2^. Geologically, the study area is characterized by limestone bedrock, steep to near-vertical limestone cliffs, and shallow, nutrient-deficient soils. The vegetation is predominantly evergreen forest, comprising herbs, shrubs, and small to large trees. The average annual rainfall and temperature are 2162 mm and 27.64 °C, respectively, as reported by the Hydro Informatics Institute and the Ministry of Energy, Thailand. The elevation of the study area ranges from 50 to 320 m above sea level. Three survey routes, each approximately 50 m wide and covering a combined area of approximately 0.25 km^2^, were established to represent the major habitat conditions and levels of human disturbance within the study area: a foothill route (Route 1; 600 m long), a limestone hill route (Route 2; 300 m long), and a roadside route adjacent to limestone hills (Route 3; 4000 m long) (Figure 6). Repeated surveys across the three routes were conducted to maximize the detection of Fabaceae species occurring in different habitat conditions and to account for seasonal variation in species occurrence.

### 4.2. Plant Collection, Identification, and Morphological Study

Thirty-four Fabaceae samples, each with three duplicates, were collected in the field, and were then dried, pressed, and deposited at the QBG herbarium. Plant characteristics, including habit, height, flower color, fruit color, and others, were recorded during fieldwork. Ecological and geographical information was documented, and photographs were taken. Plant identification was primarily based on the *Flora of Thailand* [7,8,9] and relevant published articles [10,11,12,13,14,15,16,17,18]. Identifications were further verified through comparison with type specimens and original species descriptions. In addition, morphological features of each species were examined using both recent field collections and specimens kept at the QBG herbarium. The herbarium acronym used in this study follows Thiers [51].

### 4.3. Leaf Epidermal Study

Twenty-three species were sampled for leaf epidermal study and voucher specimens were provided in Table 1. These species were selected as representatives of each genus for this study. Fully mature leaves from 23 collections were selected as the fifth leaf from the shoot apex. One leaf was sampled from each of the three duplicates per collection, resulting in a total of three leaves per collection. Leaf samples were fixed in 70% ethanol. Three areas on adaxial and abaxial epidermis of each leaf were peeled using a razor blade and gently brushed with a paintbrush. The epidermis was then cleared with 10% Clorox, washed with tap water, and stained with 1% Safranin O. The stained epidermis, measuring approximately 5 × 5 mm, was mounted on a microscopic slide, examined throughout the entire area under a light microscope (OLYMPUS BX43, Olympus Cooperation, Tokyo, Japan) to capture the range of epidermal characteristics and variation, and photographed using OLYMPUS cellSens Standard software ver. 1.18. Terminology for epidermal features mainly follows Metcalfe and Chalk [52] and Zhao et al. [35]. Ten stomata of each leaf surface were measured using the ImageJ program v.1.51n http://imagej.nih.gov/ij (18 July 2026) and stomatal size was calculated using a modified method described by Taylor et al. [53] as follows:

Stomatal size (μm^2^) = guard cell length × ∑guard cell widths

Three microscopic fields were examined for each tissue sample, and the numbers of stomata and epidermal cells were counted in each field. The stomatal index (SI) was calculated for each field using the formula of Metcalfe and Chalk [52]:

SI = (S/(E + S)) × 100

where SI is the stomatal index, S is the number of stomata per unit area, and E is the number of epidermal cells in the same area.

## 5. Conclusions

This study provides baseline data on the diversity of Fabaceae in a limestone area where 23 genera and 30 species were recorded. The morphological and leaf epidermal characteristics documented here provide taxonomic and systematic information. A limitation of this study is the geographically focused sampling, which may not capture the full extent of Fabaceae diversity and variation in leaf epidermal characteristics across other limestone habitats. Nevertheless, these findings provide an important baseline for future research on the taxonomy, ecology, and distribution of Fabaceae in limestone ecosystems, particularly through broader geographic sampling. The occurrence of endemic and threatened plant species highlights the conservation importance of limestone habitats and provides evidence for prioritizing these areas for biodiversity conservation and habitat protection. However, the conservation value of these habitats is increasingly challenged by human disturbances, which may threaten plant communities and biodiversity, particularly vulnerable, endemic, and rare species confined to limestone ecosystems.

## Figures and Tables

**Table 1:** Fabaceae found in the Thung Song limestone area in southern Thailand, with ecological data, phenology, conservation status, and specimens examined. Superscript ^a^ indicates voucher specimens for anatomical study. Conservation status follows Larsen et al. [7] and Chamchumroon et al. [28].

Species [Conservation Status]	Habitat	Flowering	Specimens Examined
**Subfamily Caesalpinioideae**			
1. *Adenanthera pavonina* L.	Ev	January–July	Manorin et al. 31 (QBG) ^a^, Srisanga 4630 (QBG)
2. *Biancaea parviflora* (Prain) Mayta & Molinari	Ev	October–January	Manorin et al. 39 (QBG) ^a^ & 40 (QBG)
3. *Entada rheedei* Spreng.	Ro, Ag	December–May	Manorin et al. 36 (QBG) ^a^, Norsaengsri & Tathana 7900 (QBG), Norsaensri 3609 (QBG)
4. *Guilandina major* (Medik.) Small	Ro, Ag	September–February	Manorin et al. 34 (QBG) ^a^
5. *Leucaena leucocephala* (Lam.) de Wit *	Ro, Ag	January–December	Manorin et al. 41 (QBG) ^a^, Balslev et al. 9578 (QBG)
6. *Mimosa diplotricha* C.Wright *	Ro, Ag	November–July	Manorin et al. 20 (QBG) ^a^
7. *M. pudica* L. *	Ro, Ag	January–December	Manorin et al. 4 (QBG)
8. *Senegalia megaladena* (Desv.) Maslin, Seigler & Ebinger	Ro, Ag	February–December	Manorin et al. 35 (QBG), Balslev et al. 9444 (QBG)
9. *S. rugata* (Lam.) Britton & Rose	Ro, Ag	November–April	Manorin et al. 24 (QBG), La-ongsri et al. 2794 (QBG)
10. *S. torta* (Roxb.) Maslin, Seigler & Ebinger	Ev	October	Manorin et al. 6 (QBG) ^a^
11. *Senna timoriensis* (DC.) H.S.Irwin & Barneby	Ro, Ag	June–March	Manorin et al. 10 (QBG) & 23 (QBG)
12. *S. tora* (L.) Roxb. *	Ro, Ag	January–December	Manorin et al. 15 (QBG) ^a^
**Subfamily Cercidoideae**			
13. *Bauhinia pottsii* G.Don var. *subsessilis* (Craib) de Wit	Ro, Ag	September–May	Manorin et al. 9 (QBG) ^a^
14. *Phanera tubicalyx* (Craib) Bandyop. & Ghoshal [EN, VU]	Ev	March–April	Manorin et al. 38 (QBG) ^a^
15. *Phanera* sp.	Ev	-	Manorin et al. 7 (QBG)
**Subfamily Papilionoideae**			
16. *Aeschynomene americana* L. *	Ro, Ag	January–December	Manorin et al. 11 (QBG) ^a^
17. *Alysicarpus vaginalis* (L.) DC.	Ro, Ag	January–December	Manorin et al. 21 (QBG) ^a^
18. *Calopogonium mucunoides* Desv. *	Ro, Ag	October–April	Manorin et al. 22 (QBG) ^a^
19. *Canavalia cathartica* Thouars	Ro, Ag	May–February	Manorin et al. 13 (QBG) ^a^
20. *Centrosema molle* Mart. ex Benth. *	Ro, Ag	October–April	Manorin et al. 1 (QBG) ^a^
21. *Derris elliptica* (Wall.) Benth.	Ro, Ag	January–June	Manorin et al. 33 (QBG) ^a^
22. *Flemingia macrophylla* (Willd.) Kuntze ex Merr.	Ro, Ag	November–June	Manorin et al. 2 (QBG) & 17 (QBG)
23. *F. strobilifera* (L.) W.T.Aiton [VU]	Ro, Ag	November–June	Manorin et al. 3 (QBG) ^a^ & 12 (QBG)
24. *Grona heterophylla* (Willd.) H.Ohashi & K.Ohashi	Ro, Ag	January–December	Manorin et al. 16 (QBG)
25. *G. triflora* (L.) H.Ohashi & K.Ohashi	Ro, Ag	January–December	Manorin et al. 14 (QBG) ^a^
26. *Macroptilium lathyroides* (Benth.) Urb. *	Ro, Ag	January–December	Manorin et al. 32 (QBG) ^a^
27. *Mucuna stenoplax* Wilmot-Dear [R]	Ro, Ag	October–April	Manorin et al. 37 (QBG) ^a^
28. *Neustanthus phaseoloides* (Roxb.) Benth.	Ro, Ag	September–March	Manorin et al. 19 (QBG) ^a^
29. *Pleurolobus gangeticus* (L.) J.St.-Hil. ex H.Ohashi & K.Ohashi	Ro, Ag	October–March	Manorin et al. 8 (QBG) & 18 (QBG) ^a^
30. *Teramnus labialis* (L.f.) Spreng.	Ev	October–March	Manorin et al. 5 (QBG) ^a^

Note: - = no data, * = introduced species, Ag = agricultural area, EN = endemic, Ev = evergreen forest, R = rare species, Ro = roadside, VU = vulnerable.

**Table 2:** Leaf epidermal characters of 23 species of Fabaceae in the Thung Song limestone area in southern Thailand. **Ad** is adaxial epidermis and **Ab** is abaxial epidermis.

Species	Cell Shape		Anticlinal Wall Type		Stomatal Type		Stomatal Size (Mean ± S.D., μm ^2^)		Stomatal Index (Mean ± S.D., %)		Trichome Type	
	Ad	Ab	Ad	Ab	Ad	Ab	Ad	Ab	Ad	Ab	Ad	Ab
1. *Adenanthera pavonina*	Ji	Ji	Si	Si	-	Pa	-	343.76 (±14.14)	*	13.69 (±0.86)	-	-
2. *Aeschynomene americana*	Ji, Ir	Ji, Ir	Un	Un	La	La	163.67 (±10.09)	214.38 (±8.29)	18.02 (±0.74)	23.86 (±0.16)	-	-
3. *Alysicarpus vaginalis* ^1^	Po	Po	St	St	Am, La, Pa	Am, La, Pa	297.21 (±49.68)	162.43 (±7.25)	13.09 (±2.97)	18.74 (±1.14)	Ca	Ho, Uc, Ca
4. *Bauhinia pottsii* var. *subsessilis*	Ir	Ji, Ir	Un	Un	Am, La, Pa	Am, La, Pa	201.22 (±45.86)	179.38 (±0.62)	0.85 (±0.78)	15.76 (±0.80)	Mu	Mu
5. *Biancaea parviflora*	Ji	Ji, Ir	Sn	Un	-	An	-	187.52 (±17.13)	*	10.90 (±0.58)	-	-
6. *Calopogonium mucunoides*	Ji	Ji	Si	Si	Am, La	Am, La, Pa	441.1 (±31.54)	381.3 (±20.25)	12.38 (±1.14)	23.46 (±1.41)	Uc, Ca	Uc, Ca
7. *Canavalia cathartica* ^3^	Ji, Ir	Ir	Un	Un	Am, La	Am, La, Pa	553.3 (±122.36)	514.29 (±63.75)	3.41 (±1.97)	15.46 (±1.78)	Uc, Ca	Uc, Ca
8. *Centrosema molle*	Ji	Ji	Si	Si	La	La	173.67 (±5.80)	152.4 (±38.54)	3.42 (±2.64)	20.46 (±0.90)	Uc, Ca	Uc, Ca
9. *Derris elliptica*	Ji, Ir	Ji, Ir	Un	Sn, Un	-	La, Pa	-	292.37 (±21.63)	*	14.78 (±1.86)	-	Uc
10. *Entada rheedei*	Po	Ir, Po	St	St, Un	-	Pa	-	495.62 (±52.25)	*	17.84 (±1.61)	-	-
11. *Flemingia strobilifera*	Ji	Ji	Si	Si	La	Am, La	257.91 (±48.32)	241.86 (±33.34)	3.18 (±1.48)	17.98 (±1.86)	Ca	Uc, Ca
12. *Grona trifloral* ^1^	Ji	Ji, Ir	Un	Un	La	Am, Pa, La	114.63 (±17.57)	143.88 (±0.68)	12.14 (±1.34)	13.67 (±1.15)	-	Uc, Ca, Ho
13. *Guilandina major*	Ir, Po	Ji, Ir	St, Un	Un	-	Am	-	608.88 (±16.42)	*	7.26 (±0.60)	-	Uc
14. *Leucaena leucocephala*	Ji, Ir	Ji, Ir	Un	Un	Am, La	Am, La, Pa	400.84 (±39.51)	519.35 (±67.52)	4.16 (±2.46)	12.44 (±2.88)	-	-
15. *Macroptilium lathyroides*	Ji, Ir	Ji, Ir	Un	Si, Un	Am, La	Am, La, Pa	319.89 (±9.50)	353.59 (±56.91)	13.99 (±1.09)	21.05 (±1.82)	-	Uc, Ca
16. *Mimosa diplotricha*	Ji	Ji	Si	Si	Am, La, Pa	Am, La, Pa	175.83 (±10.31)	207.64 (±12.42)	3.61 (±4.23)	17.27 (±1.59)	Uc	Uc
17. *Mucuna stenoplax*	Ji	Ji	Si	Si	-	Am, La, Pa	-	494.06 (±62.78)	*	14.42 (±3.41)	Ca	Uc, Ca
18. *Neustanthus phaseoloides*	Ji	Ji	Si	Si, Un	Am, La, Pa	Am, La, Pa	385.83 (±29.93)	324.74 (±46.52)	9.93 (±0.60)	21.86 (±1.76)	Uc, Ca	Uc, Ca
19. *Phanera tubicalyx* ^2^	Ji, Po	Po	St, Sn	St, Un	-	Pa	-	338.72 (±40.72)	*	12.88 (±0.65)	-	-
20. *Pleurolobus gangeticus*	Ji, Ir	Ji, Ir	Si, Un	Un	-	Am, La	-	207.2 (±3.73)	*	20.08 (±3.73)	Uc, Ho	Uc, Ca
21. *Senegalia torta*	Ji	Ji	Si	Si	-	La, Pa	-	226.86 (±13.56)	*	9.35 (±1.27)	Uc	Uc
22. *Senna tora*	Po	Ir, Po	St	St, Un	La	La, Pa	358.04 (±26.13)	381.93 (±9.98)	14.88 (±3.66)	18.88 (±0.24)	-	Mu
23. *Teramnus labialis*	Ji	Ji	Si	Si, Un	-	Am, La, Pa	-	348.48 (±23.71)	*	10.60 (±1.36)	Uc, Ca	Uc, Ca

Note: - = absent, * = stomata absent and then stomatal index not calculated, Am = anomocytic, An = anisocytic, Ca = capitate glandular, Ho = hooked, Ir = irregular, Ji = Jigsaw-liked, La = laterocytic, Mu = multicellular uniseriate, Pa = paracytic, Po = polygonal, Si = sinuate, Sn = sinuolate, St = straight arched, Uc = unicellular, Un = undulate. Each superscript at some taxon names indicated the characteristics as follows: ^1^ taxon with single papillae on outer periclinal wall of epidermal cells, ^2^ taxon with multiple papillae on outer periclinal wall of epidermal cells, and ^3^ taxon with peculiar paired crystals in epidermal cells.

## Data Availability

All relevant data are included in the manuscript.

## Supplementary Materials

The following supporting information can be downloaded at: https://www.mdpi.com/article/10.3390/plants15172605/s1, Table S1: Morphological characteristics of Fabaceae from Thung Song limestone area in southern Thailand.
