# Supplementary material for: Diversity, Leaf Anatomy, and Conservation of Fabaceae in a Thai Limestone Area
Source: Plants (Basel). 2026 Aug 26;15(17):2605. doi: 10.3390/plants15172605 (PMC13567078; doi:10.3390/plants15172605)
Supplement: Supplementary file 1 [file plants-15-02605-s001.zip › plants-4476368-supplementary.pdf]

plants

MDPI

Supplementary Table S1. Morphological characteristics of Fabaceae from Thung Song limestone area in southern Thailand. Superscript [1] indicated species with spine or prickle and [2] indicated species with tendril.

Species	Morphological characters									
	Habit	Leaf		Flower						Fruit type
		Type	Gland	Symmetry	Sepal type	Petal type	Petal number	Stamen type	Stamen number	
**Subfamily Caesalpinioideae**										
1. *Adenanthera pavonina*	Tr	Bi	-	Ra	Un	Fr	5	Fr	10	De
2. *Biancaea parviflora*[1]	Sc	Bi	-	Bs	Fr	Fr	5	Fr	10	De
3. *Entada rheedei*[2]	Cl	Bi	-	Ra	Un	Fr	5	Fr	10	Ts
4. *Guilandina major*[1]	Sc	Bi	-	Bs	Fr	Fr	5	Fr	10	De
5. *Leucaena leucocephala*	Tr	Bi	+	Ra	Fr	Fr	5	Fr	10	De
6. *Mimosa diplotricha*[1]	He	Bi	+	Ra	Un	Un	4	Fr	8	Ts
7. *M. pudica*[1]	He	Bi	+	Ra	Un	Un	4	Fr	4	Ts
8. *Senegalia megaladena*[1]	Sc	Bi	+	Ra	Un	Un	5	Fr	>15	De
9. *S. rugata*[1]	Sc	Bi	+	Ra	Un	Un	5	Fr	>15	Ts
10. *S. torta*[1]	Cl	Bi	+	Ra	Un	Un	5	Fr	>15	De
11. *Senna timoriensis*	Tr	Pi	-	Bs	Fr	Fr	5	Fr	10	De
12. *S. tora*	He	Pi	+	Bs	Fr	Fr	5	Fr	7	De
**Subfamily Cercidoideae**										
13. *Bauhinia pottsii* var. *sub-sessilis*	Tr	Si	-	Bs	Un,Fr	Fr	5	Fr	3[2]	De
14. *Phanera tubicalyx*[2]	Cl	Si	-	Bs	Un	Fr	5	Fr	3[7]	*
15. *Phanera* sp.[2]	Cl	Si	-	Bs	*	*	*	*	*	*
**Subfamily Papilionoideae**										
16. *Aeschynomene americana*	He	Pi	-	Bs	Un	Fr,Un	5	Un	10	Ts
17. *Alysicarpus vaginalis*	He	Ul	-	Bs	Un	Fr,Un	5	Un	10	Ts
18. *Calopogonium mucunoides*	Cl	Tl	-	Bs	Un	Fr,Un	5	Un	10	De
19. *Canavalia cathartica*	Cl	Tl	-	Bs	Un	Fr,Un	5	Un	10	De
20. *Centrosema molle*	Cl	Tl	-	Bs	Un	Fr,Un	5	Un	10	De
21. *Derris elliptica*	Cl	Pi	-	Bs	Un	Fr,Un	5	Un	10	In
22. *Flemingia macrophylla*	Sh	Tl	-	Bs	Un	Fr,Un	5	Un	10	De
23. *F. strobilifera*	Sh	Ul	-	Bs	Un	Fr,Un	5	Un	10	De
24. *Grona heterophylla*	He	Tl	-	Bs	Un	Fr,Un	5	Un	10	De,Ts
25. *G. triflora*	He	Tl	-	Bs	Un	Fr,Un	5	Un	10	De,Ts
26. *Macroptilium lathyroides*	He	Tl	-	Ir	Un	Fr,Un	5	Un	10	De
27. *Mucuna stenoplax*	Cl	Tl	-	Bs	Un	Fr,Un	5	Un	10	De
28. *Neustanthus phaseoloides*	Cl	Tl	-	Bs	Un	Fr,Un	5	Un	10	De
29. *Pleurolobus gangeticus*	He	Ul	-	Bs	Un	Fr,Un	5	Un	10	De,Ts
30. *Teramnus labialis*	Cl	Tl	-	Bs	Un	Fr,Un	5	Un	10	De

Note: - = absent, + = present, * = no data, Bi = bipinnately compound leaf, Bs = bilateral symmetry, Cl = climber, De = dehiscent, Fr = free, He = herb, In = indehiscent, Ir = irregular, Pi = pinnately compound leaf, Ra = radial symmetry, Sc = scandent, Sh = shrub, Si = simple leaf, Tr = tree, Tl = trifoliolate leaf, Ts = transverse splitting, Ul = unifoliolate leaf, Un = united. Data in a square bracket ([ ]) provide the number of staminodes.

https://doi.org/10.3390/plants15172605
